# Bidirectional regulation of synaptic SUMOylation by Group 1 metabotropic glutamate receptors

**DOI:** 10.1007/s00018-022-04405-z

**Published:** 2022-06-23

**Authors:** Marie Pronot, Gwénola Poupon, Lara Pizzamiglio, Marta Prieto, Isabel Chato-Astrain, Iliona Lacagne, Lenka Schorova, Alessandra Folci, Frédéric Brau, Stéphane Martin

**Affiliations:** 1 Université Côte d’Azur, CNRS, IPMC, Valbonne, France; 2 Université Côte d’Azur, INSERM, CNRS, IPMC, Valbonne, France; 3 Institut de Pharmacologie Moléculaire et Cellulaire, UMR7275, Centre National de la Recherche Scientifique, Université Côte d’Azur, 660 route des lucioles, 06560 Valbonne, France

**Keywords:** SUMO, deSUMOylation, SENP1, Synapse, PKC, CaMKII, mGlu1, mGlu5 receptors

## Abstract

SUMOylation is a post-translational modification essential to cell homeostasis. A tightly controlled equilibrium between SUMOylation and deSUMOylation processes is also critical to the neuronal function including neurotransmitter release and synaptic transmission and plasticity. Disruption of the SUMOylation homeostasis in neurons is associated with several neurological disorders. The balance between the SUMOylation and deSUMOylation of substrate proteins is maintained by a group of deSUMOylation enzymes called SENPs. We previously showed that the activation of type 5 metabotropic glutamate receptors (mGlu5R) first triggers a rapid increase in synaptic SUMOylation and then upon the sustained activation of these receptors, the deSUMOylase activity of SENP1 allows the increased synaptic SUMOylation to get back to basal levels. Here, we combined the use of pharmacological tools with subcellular fractionation and live-cell imaging of individual hippocampal dendritic spines to demonstrate that the synaptic accumulation of the deSUMOylation enzyme SENP1 is bidirectionally controlled by the activation of type 1 mGlu1 and mGlu5 receptors. Indeed, the pharmacological blockade of mGlu1R activation during type 1 mGluR stimulation leads to a faster and greater accumulation of SENP1 at synapses indicating that mGlu1R acts as a brake to the mGlu5R-dependent deSUMOylation process at the post-synapse. Altogether, our findings reveal that type 1 mGluRs work in opposition to dynamically tune the homeostasis of SUMOylation at the mammalian synapse.

**Supplementary Information:**

The online version contains supplementary material available at 10.1007/s00018-022-04405-z.

## Introduction

Neuronal transmission takes place at highly specialized structures called synapses. Post-translational modifications (PTMs) play an essential role in synaptic communication by dynamically regulating protein–protein interactions at both pre- and postsynaptic sites. In the past decade, the PTM SUMOylation has emerged as an important regulator of the neuronal and synaptic functions [1, 2]. SUMOylation consists in the covalent, but reversible conjugation of the Small Ubiquitin-like MOdifier (SUMO1-3) polypeptides (~ 100 amino acids; ~ 11 kDa) to specific lysine residues of substrate proteins [3, 4]. It participates in the dynamic regulation of multi-protein complexes by creating new binding sites for specific interactors or alternatively, by disrupting or preventing protein–protein interactions [5]. SUMOylation regulates the function of several extranuclear proteins involved in neuronal excitability [6–8], postsynaptic differentiation [9] as well as in synaptic transmission and plasticity [10–12].

A tight equilibrium between SUMOylation and deSUMOylation is constantly maintained and essential in order for the SUMO-targeted proteins to shape their molecular networks and inherently, to fulfil their function. This balance is preserved by the coordinated action of a dedicated pathway by which SUMO is conjugated to their substrates via the sole E2-conjugating enzyme Ubc9 with or without the need of an E3 SUMO-ligase. SUMO can then be quickly removed by specific deSUMOylation enzymes called Sentrin-specific proteases (SENPs). There are six different SENPs in mammals that are essentially characterized by their selectivity for the SUMO paralogs and/or their subcellular localisation [13]. For instance, SENP1 is partly localized to dendritic spines [14–18] and can efficiently remove SUMO moieties from target proteins [2, 16].

The homeostasis of protein SUMOylation must be preserved to maintain the synaptic function. Importantly, alterations in the SUMOylation/deSUMOylation balance are directly correlated with the development of neurodevelopmental and neurodegenerative diseases, including, but not restricted to, the Rett syndrome or Parkinson’s and Alzheimer’s diseases [1, 2]. We previously reported the existence of a spatiotemporal and activity-dependent regulation of the SUMOylation process in the brain [7, 15]. In addition, we demonstrated that the activation of the metabotropic glutamate mGlu5 receptor promotes the transient trapping of Ubc9 in dendritic spines leading to a rapid increase in synaptic SUMOylation [7]. More recently, we showed that the activation of type 1 mGluRs participates in the redistribution of SENP1 to post-synaptic sites by decreasing the exit rate of the deSUMOylation enzyme from dendritic spines and consequently, reducing synaptic SUMOylation to basal levels [16]. However, the precise mechanisms and signaling pathways driving the spatiotemporal redistribution of the SENP enzymes at synapses are still not known, but of critical importance to better understand how the levels of synaptic SUMOylation is locally maintained.

Here, we identified a bidirectional role of type 1 mGlu1 and mGlu5 receptors on the activity-dependent accumulation of the deSUMOylating enzyme SENP1 in dendritic spines using a combination of biochemical and live cell-imaging approaches. We show that the mGlu5R activation is primarily responsible for the accumulation of SENP1 in dendritic spines of rat cultured hippocampal neurons whereas the activation of mGlu1R rather acts as a brake to this accumulation. This activity-dependent accumulation at post-synaptic sites requires the activation of either the Protein Kinase C (PKC) or the Ca^2+^/calmodulin-dependent protein kinase II (CaMKII) downstream of the mGluRs. Altogether, our data clearly demonstrate that type 1 mGlu1 and mGlu5 receptors act in opposition to finely tune the synaptic levels of SENP1 and dynamically maintain the level of SUMOylation at synapses.

## Results

### The synaptic accumulation of SENP1 is triggered by the activation of mGlu5R

**Fig. 1:**
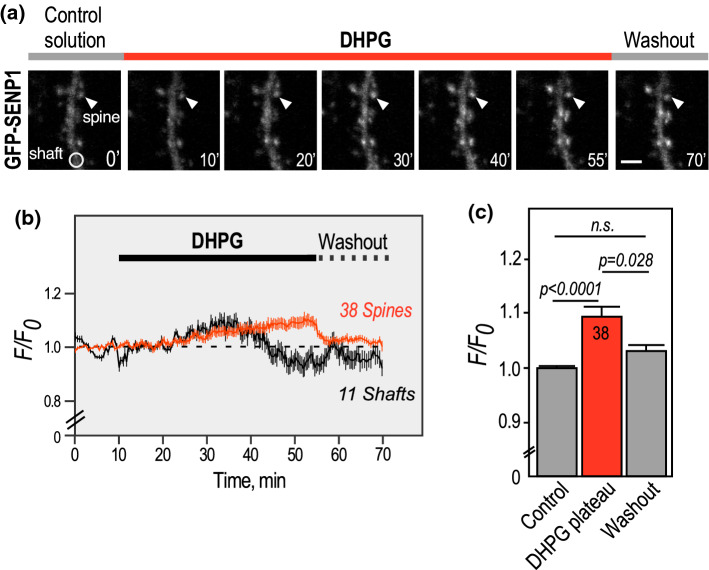
Activity-dependent redistribution of GFP-SENP1 into spines. **a** Representative confocal images of a time-lapse recording of a GFP-SENP1 expressing rat hippocampal secondary dendrite in control and DHPG (50 µM)-stimulated conditions as indicated. Scale bar, 5 µm. **b** Quantification of time lapse experiments showing the variation of normalized fluorescence intensity ± SEM in spines (*n* = 38) and shafts (*n* = 11). **c** Histograms showing the mean GFP-SENP1 fluorescence intensity ± SEM in spines in control, DHPG plateau (50–55’; 1.094 ± 0.021) and washout (1.028 ± 0.010). Statistics: One-way ANOVA with Tukey post hoc test. P values are indicated on the bars. n.s., non-significant

The post-synaptic targeting of several enzymes important for the synaptic function is triggered by neuronal activity [7, 15, 16, 19, 20]. To assess how neuronal activity regulates the synaptic accumulation of the deSUMOylation enzyme SENP1, we expressed a GFP-tagged version of SENP1 [16] in rat hippocampal neurons and combined time-lapse microscopy with the use of (S)-3,5-Dihydroxyphenylglycine (DHPG) to activate both type 1 mGlu1 and mGlu5 receptors (Fig. 1). Using live-cell imaging, we recorded the redistribution of GFP-SENP1 into dendritic spines in real time during the mGluR stimulation. Activation of type 1 mGluRs with DHPG led to a slow but significant increase in GFP-SENP1 fluorescence in dendritic spines, reaching a plateau 40 min after the beginning of the drug application (Fig. 1; Supplementary video 1). In parallel, we monitored a decrease in GFP-SENP1 fluorescence in the dendritic shaft as soon as the plateau of GFP-SENP1 fluorescence was reached (Fig. 1a, b). The synaptic GFP-SENP1 fluorescence accumulated in dendritic spines returned to initial levels when the DHPG was exchanged for the control solution, indicating that the type 1 mGluR-dependent accumulation of GFP-SENP1 into the post-synaptic compartments is reversible (Fig. 1; Supplementary video 1). We also verified that both the overall fluorescence and synapto-dendritic localisation of GFP-SENP1 remain unchanged in basal unstimulated conditions over the time course of these experiments (Supplementary Fig. 1).

Importantly, the gradual accumulation of GFP-SENP1 at post-synaptic sites measured upon the sustained activation of type 1 mGluRs results from the concomitant activation of both mGlu1 and mGlu5 receptors in the presence of DHPG. Type I mGluRs are G-protein coupled receptors that trigger distinct signaling pathways by activating different kinases [21]. Thus, we assessed the effects of the separate activation of each receptor subtype on downstream signaling pathways by investigating the levels of MAP kinase ERK phosphorylation **(**Supplementary Fig. 2). As expected, an increase in ERK phosphorylation was triggered upon the incubation of hippocampal neurons with DHPG [22]. This increase in ERK phosphorylation was fully blocked when the neurons were preincubated with the specific mGlu5R antagonist MPEP, but not when neurons were pretreated in the presence of the potent non-competitive mGlu1R antagonist JNJ16259685 [23]. These data indicate that the activation of these receptors differentially promotes ERK phosphorylation, with mGlu5Rs being the sole activator of this signaling cascade in rat cultured hippocampal neurons.

**Fig. 2:**
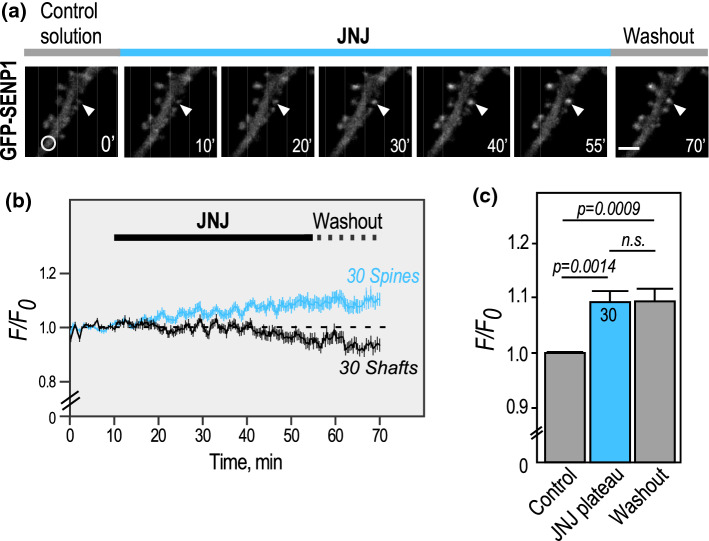
The constitutive activity of mGlu1Rs participates in the steady state regulation of the synaptic SENP1 content. **a** Representative confocal images of a time-lapse recording of a GFP-SENP1 expressing rat hippocampal secondary dendrite in the presence of the mGlu1R antagonist JNJ16259685. Scale bar, 5 µm. **b** Quantification of time lapse experiments showing the variation of normalized fluorescence intensity ± SEM in spines (*n* = 30) and shafts (*n* = 30). **c** Histograms showing the mean GFP-SENP1 fluorescence intensity ± SEM in spines in control, plateau (50–55’; 1.092 ± 0.020) and washout (1.094 ± 0.022). Statistics: One-way ANOVA with Tukey post hoc test. P values are indicated on the bars. *n.s.* non-significant

Since type 1 mGluRs also present a constitutive activity [24], we first used time-lapse microscopy to assess the synaptic accumulation of GFP-SENP1 while blocking mGlu1R constitutive activity with the selective non-competitive mGlu1R antagonist JNJ16259685 in absence of agonist stimulation **(**Fig. 2). Upon the administration of JNJ16259685, we measured a slow but significant increase in GFP-SENP1 fluorescence in dendritic spines, reaching a plateau about 40 min after the beginning of the antagonist application (Fig. 2; Supplementary video 2). This synaptic accumulation was mirrored by a decrease in GFP-SENP1 fluorescence in the dendritic shaft, indicating that the constitutive activity of mGlu1Rs participates in the regulation of the steady state levels of SENP1 in dendritic spines.

**Fig. 3:**
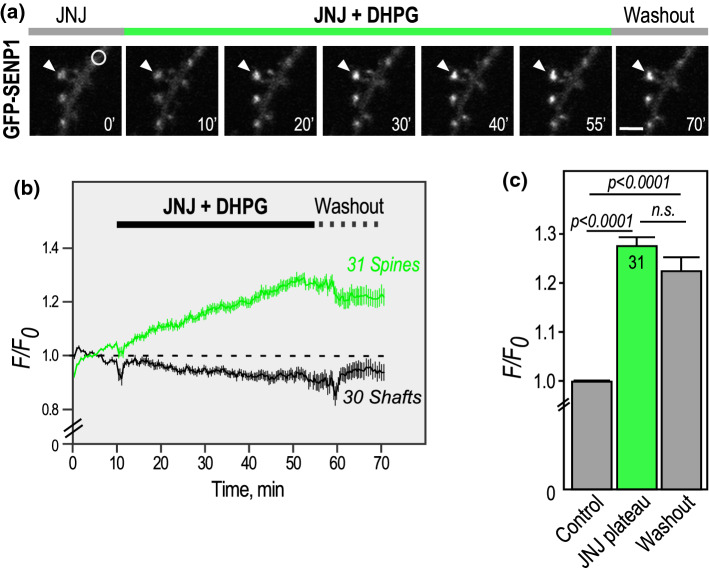
Blocking mGlu1R activation during type 1 mGluR stimulation leads to a sharp accumulation of GFP-SENP1 in dendritic spines. **a** Representative confocal images of a time-lapse recording of a GFP-SENP1 expressing rat hippocampal secondary dendrite in JNJ16259685 + DHPG-stimulated conditions as indicated. Scale bar, 5 µm. **b** Quantification of time-lapse experiments showing the variation of normalized fluorescence intensity ± SEM in spines (*n* = 31) and shafts (n = 30). **c** Histograms showing the mean GFP-SENP1 fluorescence intensity ± SEM in spines in control, plateau (50–55’; 1.277 ± 0.018) and washout (1.223 ± 0.024). Statistics: One-way ANOVA with Tukey post hoc test. P values are indicated on the bars. n.s., non-significant

To further explore the contribution of each type 1 mGluR subtype in the activity-dependent accumulation of SENP1 into dendritic spines, we measured the impact of the mGlu1R antagonist JNJ16259685 in the presence of the type 1 mGluR agonist DHPG (Fig. 3; Supplementary video 3). Surprisingly, time-lapse recordings of the synaptic targeting of GFP-SENP1 in the presence of both DHPG and JNJ16259685 revealed that the accumulation of GFP-SENP1 into spines is far much faster and reaches higher levels when the activation of mGlu1R is prevented. Indeed, the levels of synaptic GFP-SENP1 fluorescence accumulation were almost three times higher than with DHPG alone (Fig. 1; DHPG plateau (50–55’; 1.094 ± 0.021) Vs [DHPG + JNJ16259685] plateau (50–55’; 1.277 ± 0.018). These data therefore indicate that the redistribution of SENP1 to dendritic spines is oppositely regulated by type 1 mGluRs with the activation of mGlu5Rs being responsible for addressing SENP1 to dendritic spines, whereas mGlu1R rather acts as a brake to this post-synaptic accumulation.

### Synaptic levels of endogenous SENP1 are significantly increased when mGlu1R activation is prevented

**Fig. 4:**
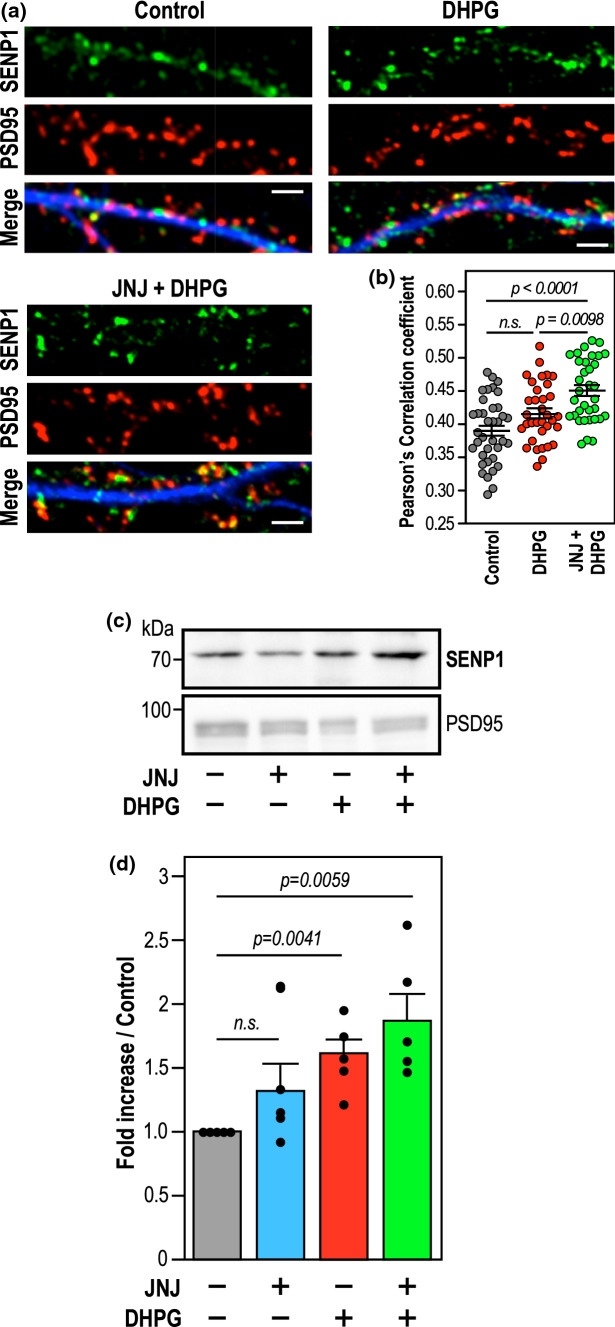
Blockade of mGlu1R activation during type 1 mGluR stimulation leads to an accumulation of endogenous SENP1 in dendritic spines. **a** Immunolabelling of fixed primary hippocampal neurons for SENP1 and PSD95 in control, DHPG (40 min) and JNJ + DHPG (40 min) conditions. Scale bar, 5 µm. **b** Quantitative representation ± SEM of control-normalized colocalization of SENP1 within PSD-95 area (Pearson’s Correlation coefficient: control (0.389 ± 0.008), DHPG (0.416 ± 0.0077), DHPG + JNJ16259685 (0.450 ± 0.008) from three independent cultures with dendrites of control (*n* = 37); DHPG (*n* = 34); DHPG + JNJ16259685 (*n* = 34). Statistics: Ordinary one-way ANOVA with a Tukey post hoc test. *p* values are indicated on the bars. ns, non-significant. **c** Representative SENP1 immunoblot performed on synaptosomal fractions (25 µg of proteins per lane) from control, JNJ16259685; 10 min DHPG and 10 min [DHPG + JNJ16259685]-treated P14 rat brain slices. The synaptic marker PSD95 was used as a loading control.  **d** Mean histogram ± SEM of synaptic SENP1 levels (JNJ16259685 (1.33 ± 0.21); DHPG (1.61 ± 0.12); DHPG + JNJ16259685 (1.87 ± 0.22) obtained from five independent experiments. Statistics: Ratio paired t-test between TTX and drug-treated conditions. *p* values are indicated on the bars. *n.s.* not significant

To further confirm that the sole activation of mGlu5R also leads to the post-synaptic accumulation of endogenous SENP1, we measured the extent of colocalisation between the endogenous SENP1 and the postsynaptic marker PSD95 in basal, DHPG and [DHPG + JNJ16259685]-treated conditions (Fig. 4a,b). In line with the live-imaging data, the extent of SENP1 colocalisation with PSD95-positive post-synaptic sites was significantly increased and maximum in [DHPG + JNJ16259685]-treated hippocampal neurons (Fig. 4b; Pearson’s correlation coefficient, Control (0.389 ± 0.008); DHPG (0.416 ± 0.0077); [DHPG + JNJ16259685] (0.450 ± 0.008), thus confirming that the specific activation of mGlu5Rs triggers the targeting and retention of endogenous SENP1 into spines.

To assess the central action of mGlu5R on the synaptic accumulation of the endogenous SENP1 in a more integrated system, we prepared synaptosomal fractions from post-natal day 14 (PND14) rat brain slices (Supplementary Fig. 3) and measured the synaptic levels of SENP1 following a 10-min stimulation with either JNJ16259685, DHPG or [DHPG + JNJ16259685] (Fig. 4c,d). As expected, we found that synaptosomal fractions were specifically enriched in synaptic proteins including Synapsin1, PSD95 and Homer1, and importantly, devoid of Coilin and GM130 proteins demonstrating the absence of nuclear and Golgi contaminants, respectively (Supplementary Fig. 3B). Interestingly, incubation of PND14 brain slices with DHPG led to a significant increase in the endogenous levels of SENP1 in synaptosomes. This increase was even greater when brain slices were coincubated with [DHPG + JNJ16259685] to specifically activate mGlu5Rs (Fig. 4c,d), further confirming the mGlu5R-driven targeting and accumulation of SENP1 to synapses.

### The mGlu5R-dependent accumulation of SENP1 at synapses requires the activation of either PKC or CaMKII, and to a lesser extent, PKA

**Fig. 5:**
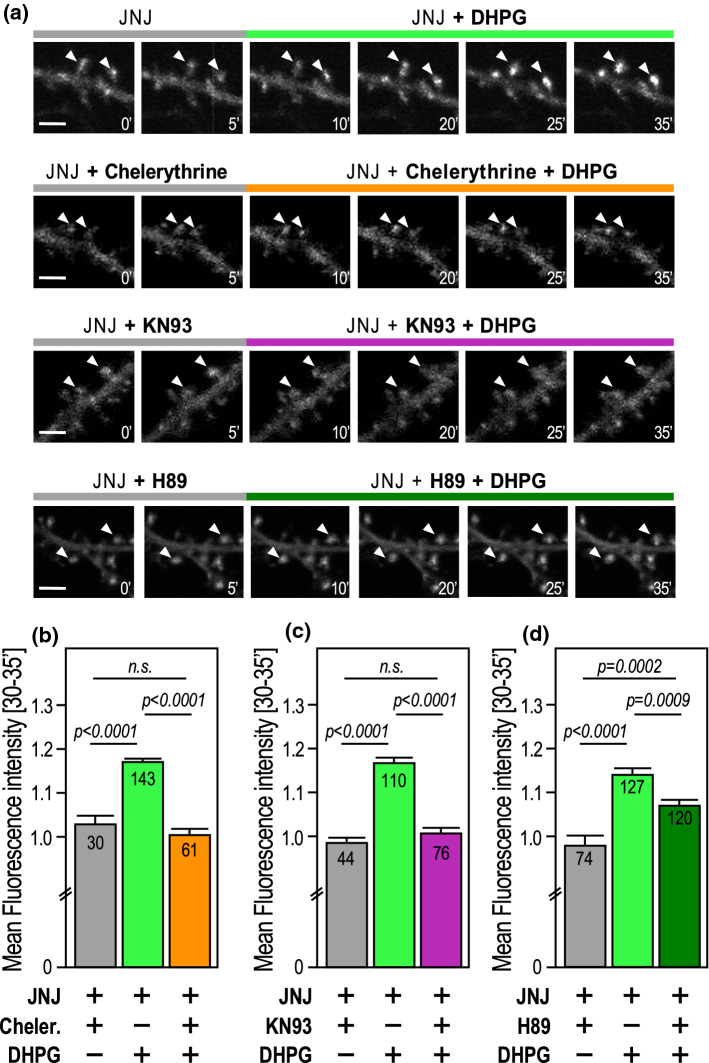
Activation of PKC or CaMKII, but not PKA, is required for the accumulation of SENP1 at synapses. **a** Representative confocal images of time-lapse recordings of GFP-SENP1-expressing rat hippocampal secondary dendrites preincubated 10 min in TTX (0.5 µM) with JNJ16259685 (0.5 µM) and either the PKC antagonist Chelerythrine (5 µM), the CaMKII antagonist KN93 (1 µM) or the PKA inhibitor H89 (1 µM) and treated in the same medium for 25 min with DHPG (50 µM) as indicated. Scale bar, 5 µm. **b** Histograms showing the mean GFP-SENP1 fluorescence intensity ± SEM in spines at the plateau (20–25 min of treatment) in control, [DHPG + JNJ16259685] (1.171 ± 0.010) and [Chelerythrine + DHPG + JNJ16259685] (1.004 ± 0.018) conditions. **c** Histograms showing the mean GFP-SENP1 fluorescence intensity ± SEM in spines at the plateau (20–25 min of treatment) in control, [DHPG + JNJ16259685] (1.168 ± 0.012) and [KN93 + JNJ16259685 + DHPG] (1.008 ± 0.012) conditions. **d** Histograms showing the mean GFP-SENP1 fluorescence intensity ± SEM in spines at the plateau (20–25 min of treatment) in control, [DHPG + JNJ16259685] (1.142 ± 0.014) and [H89 + JNJ16259685 + DHPG] (1.072 ± 0.011) conditions. Statistics: Ordinary one-way ANOVA with Tukey post hoc test. *p* values are indicated on the bars. *n.s.* non-significant

Although on different timescales, both the SUMOylation and deSUMOylation processes at synapses are controlled by the activation of type 1 mGluRs [7, 16]. However, the molecular pathways underlying this enzymatic balance are not fully understood. Type 1 mGluRs are coupled to Gαi and Gαq proteins [22]. Activation of Gαq downstream of mGlu5R stimulates PLC, resulting in the formation of intracellular inositol-1,4,5-trisphosphate (IP3) and diacylglycerol. Binding of IP3 to its intracellular receptors triggers the release of calcium ions from internal stores, and together with diacylglycerol, promote PKC activation [22]. Since the activation of PKC is required for the rapid but transient trapping of the SUMO-conjugating enzyme Ubc9 into spines [7], we questioned whether PKC activity is also important for the mGlu5R-dependent accumulation of GFP-SENP1 at post-synaptic sites. Time-lapse recordings of JNJ16259685-treated neurons stimulated with DHPG to specifically activate mGlu5Rs were preincubated with Chelerythrine, a potent inhibitor of PKC activity [25] (Fig. 5a,b; Supplementary video 4; Supplementary Figs. 4,5). Since the accumulation of GFP-SENP1 is much faster and to a larger extent when mGlu1Rs are antagonized **(**Fig. 3), we compared the levels of deSUMOylation enzymes accumulated just 20 min after the start of the DHPG incubation *(between 30 and 35’ in the experimental time sequence shown in Fig. **5**).* We demonstrated that the accumulation of synaptic GFP-SENP1 induced by the sole activation of mGlu5Rs is fully blocked when hippocampal neurons are preincubated with the PKC inhibitor Chelerythrine (Fig. 5a,b; [JNJ16259685 + DHPG] plateau 20–25’; 1.171 ± 0.010) Vs [JNJ16259685 + DHPG + Chelerythrine] plateau (20–25’; 1.004 ± 0.018). Similar results were obtained with another specific PKC inhibitor, Calphostin C (1 µM; [26]) confirming that blocking PKC activity prevents the mGlu5R-dependent accumulation of synaptic SENP1 *(data not shown)*.

The CaMKII is a serine/threonine kinase centrally involved in the synaptic function upon neuronal stimulation [20]. We thus investigated whether the activity of the CaMKII could also be involved in the post-synaptic accumulation of SENP1. We performed time-lapse recordings on GFP-SENP1-expressing hippocampal neurons pre-treated for 10 min with the potent CaMKII inhibitor KN93 [27, 28] (Fig. 5a,c; Supplementary video 4). We found that similarly to the PKC, the need of a functional CaMKII activity is required to allow the mGlu5R-dependent synaptic accumulation of GFP-SENP1 (Fig. 5a,c; [JNJ16259685 + DHPG] plateau 30–35’; 1.168 ± 0.012) Vs [JNJ16259685 + DHPG + KN93] plateau 30–35’; 1.008 ± 0.012).

Activation of Gαi downstream of mGlu5R stimulates cAMP-dependent protein kinase A (PKA) activity [22]. To evaluate the involvement of this signaling pathway on the synaptic accumulation of SENP1, we performed time-lapse recordings on GFP-SENP1 expressing hippocampal neurons pretreated for 10 min with the potent PKA inhibitor H89 (Supplementary Figs. 4,5). Preincubation with H89 reduced about half of the post-synaptic accumulation of GFP-SENP1 induced by mGlu5R activation (Fig. 5a,d; [JNJ16259685 + DHPG] plateau 30–35’; 1.142 ± 0.014) Vs [JNJ16259685 + DHPG + H89] plateau 30–35’; 1.07 ± 0.011) indicating that GFP-SENP1 accumulation is only partially blocked upon the inhibition of PKA and therefore, that PKA activation does not directly participate in the mGluR-dependent accumulation of SENP1 into dendritic spines. Altogether, these data indicate that the mGlu5R-dependent post-synaptic accumulation of GFP-SENP1 absolutely requires the activation of either PKC or CaMKII rather than PKA.

### Concurrent mGlu1R and mGlu5R regulation of synaptic SUMOylation

**Fig. 6:**
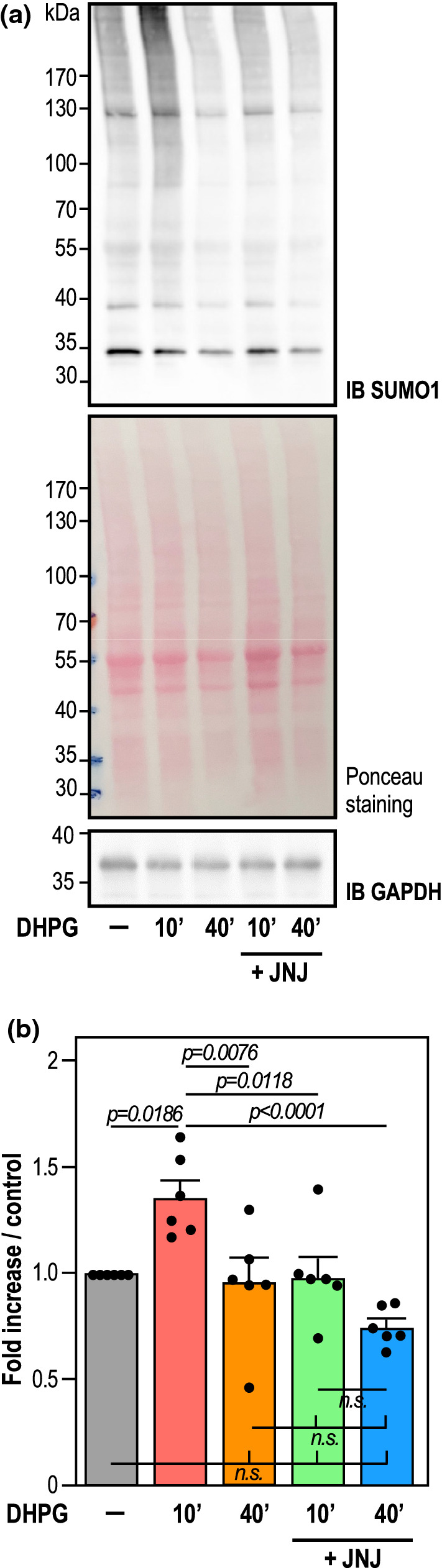
mGlu1R activity regulates SUMOylation at synapses. **a** Representative immunoblot of synaptic SUMOylated proteins from control, 10 min or 40 min DHPG and 10 min or 40 min DHPG + JNJ16259685-treated cortical neurons (20 DIV). Ponceau S staining was used as a loading/normalization control. GAPDH is also shown as an additional loading control. **b** Summary histogram shows mean ± SEM of SUMO1-modified proteins (DHPG 10 min (1.359 ± 0.078), DHPG 40 min (0.960 ± 0.110), DHPG + JNJ16259685 10 min (0.979 ± 0.093), DHPG + JNJ16259685 40 min (0.748 ± 0.037) obtained from six independent experiments. Statistics: Ordinary one-way ANOVA with a Tukey post hoc test. *p* values are indicated on the bars. *n.s.* non-significant

Given the deSUMOylating activity of SENP1, we finally assessed whether the synaptic enrichment of endogenous SENP1 upon the sole activation of mGlu5R could modulate the synaptic levels of protein SUMOylation (Fig. 6). We thus isolated crude synaptosomes from rat cortical-cultured neurons. In line with our previous studies [16], a 10-min DHPG treatment led to a significant increase in synaptic levels of SUMO1-modified proteins while 40-min DHPG treatment decreases synaptic SUMOylation to basal levels (Fig. 6). By contrast, the enrichment of SENP1 at synapses after 10 min of mGlu5R activation (Fig. 4c [JNJ16259685 + DHPG]) prevents the increase in synaptic SUMOylation that occurs in DHPG-stimulated conditions (Fig. 6). Overall, these results strongly support the bidirectional action of type1 mGluRs on the synaptic accumulation of SENP1 further highlighting the complex regulation of SUMOylation homeostasis in the brain.

## Discussion

**Fig. 7:**
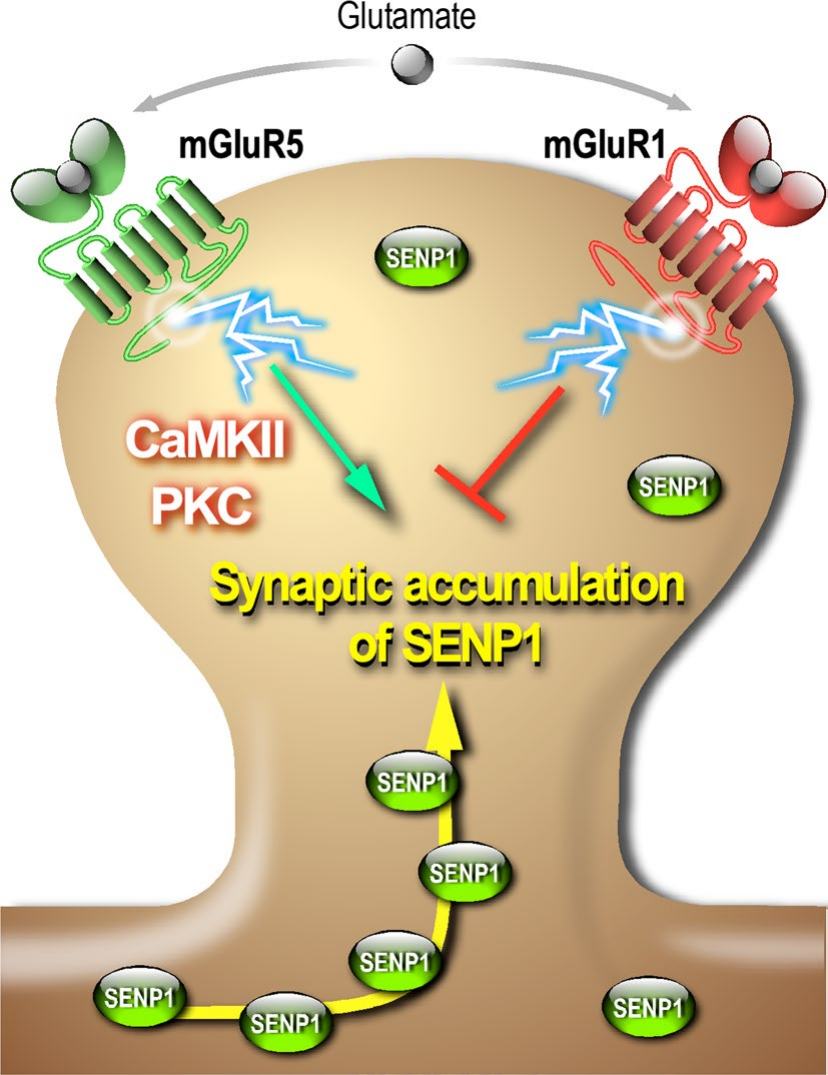
Schematic model representing the bidirectional regulation of SENP1 accumulation by type 1 mGluRs at the mammalian synapse

The synaptic balance between SUMOylation and deSUMOylation is essential to maintain a proper synaptic communication [7, 16]. Here, we demonstrated that both type 1 mGlu1 and mGlu5 receptors are involved in this dynamic regulation (Fig. 7). We showed that these receptors work in opposition to control the levels of SENP1 at the post-synapse. Indeed, the amount of SENP1 is largely increased in synapses when mGlu1Rs are blocked during a type 1 mGluR stimulation. The post-synaptic accumulation of SENP1 in response to the sole mGlu5Rs activation requires the prior activation of either PKC or CaMKII and, to a lesser extent, PKA, downstream of the mGluRs activation (Fig. 7).

The regulation of the synaptic balance between SUMOylation and deSUMOylation appears more complex than initially thought. Both these processes are regulated by the activation of mGluRs and require PKC/CaMKII activities. First, a rapid transient mGlu5R- and PKC-dependent synaptic trapping of the SUMO-conjugating enzyme Ubc9 allows the rapid SUMOylation of synaptic target proteins [7]. This is then followed by the recruitment of active deSUMOylation enzymes to get post-synaptic SUMO-modified proteins back to basal levels [16]. In this work, we provide an additional level of complexity by demonstrating that mGlu1Rs act as a brake to the mGlu5R-dependent accumulation of SENP1 enzymes at synapses. Interestingly, since glutamate binds to both type 1 mGluRs, the precise tuning of the deSUMOylation process at synapses could rely on different, but not mutually exclusive pathways. For instance, the composition of type 1 mGluRs at individual synapses could be different, with some synapses expressing more mGlu1Rs than others. Therefore, activated synapses presenting higher levels of mGlu1Rs would show less accumulation of SENP1 than synapses containing mostly mGlu5Rs. Another possibility is that the level of mGlu1/mGlu5 heterodimers could be variable between synapses, leading to distinct synaptic responses and thus, to either a delayed, or a faster accumulation of SENP1 in activated spines.

Since phosphorylation depending on either PKC or CaMKII and to a lesser extent, PKA, is essential to the synaptic accumulation of SENP1, we cannot exclude that SENP1 may also be a direct target of these kinases. In line with this hypothesis, SENP1 could readily be phosphorylated in response to neuronal activation since its primary sequence presents high predictive values for many Ser/Thr phosphorylation sites [29]. The phosphorylation of SENP1 may thus be essential to control either its activity or its subcellular targeting and/or retention in dendritic spines. Of interest is the report that SENP3, a paralog of SENP1, is phosphorylated in HeLa cells, thus preventing its deSUMOylase activity [30, 31]. Therefore, the potential activity-dependent phosphorylation of SENP1 could also inhibit its deSUMOylase activity and consequently, lead to a rapid increase in synaptic SUMOylation. Another possibility is that the activity-dependent phosphorylation of SENP1 may represent a synaptic retention cue. In this case, this modification would rather participate in the accumulation of SENP1 in mGlu5R-activated synapses in line with our data. In any case, extensive studies remain to be done to assess whether SENP1 is activity-dependently phosphorylated in response to type 1 mGluRs activation and how specific phosphorylation sites on SENP1 may exist and impact the activity and/or the synaptic redistribution of the enzyme.

The relative ratio between the two subtypes of mGluRs within individual synapses may also dictate the synaptic levels of PKC, CaMKII or PKA activity, and therefore directly impact the activity-dependent accumulation of SENP1 and the extent of SUMOylated proteins at the post-synapse. For instance, Thalhammer and colleagues reported that the increase in synaptic activity leads to the targeting of CaMKII to dendritic spines of excitatory neurons [19]. However, this post-synaptic activity-dependent targeting of the kinase is linked to the activation of NMDA receptors and, the underlying signaling pathways involve calcium mobilization and microtubule dynamics [20]. While SENP1 accumulation is not mediated by the activation of NMDAR [16], it remains to be determined whether the synaptic targeting of CaMKII is also driven by the activation of type 1 mGluRs and whether mGlu1 and mGlu5 receptors work in opposition to the synaptic accumulation of the CaMKII. In addition, CaMKII phosphorylates many synaptic proteins following neuronal activation, which is essential to the synaptic function. Therefore, we cannot rule out the possibility that the transient increase in synaptic phosphorylated proteins in activated spines could also act as a retention matrix to capture SENP1 into dendritic spines. Thus, we can assume that an interplay between these two post-translational modifications occurs in a dynamic way to spatiotemporally control the SUMOylation/deSUMOylation balance at synapses.

Interestingly, the current data also raise a potential link between brain diseases presenting defects in type 1 mGluR signaling and its potential impact on the regulation of the synaptic balance between SUMOylation and deSUMOylation. Recently, a de novo protein-truncating mutation in the *SENP1* gene has been identified in ASD patients and resulted in autistic-like behaviors in a genetic mouse model of *senp1* haploinsufficiency [32]. It is also well established that alterations of the mGlu5R-dependent function are clearly involved in the etiology of several neurological disorders, including autistic spectrum disorders, schizophrenia or chronic pain [22]. Therefore, we can postulate that a functional alteration in the mGluR function or in its downstream signaling, could also directly impact the homeostasis of protein SUMOylation/deSUMOylation by deregulating the synaptic targeting of both Ubc9 and SENP1 in activated neurons. It would thus be of interest to investigate the synapto-dendritic trafficking of Ubc9 and SENP1 in a disease context and to define whether the altered mGluR function also impairs the synaptic repertoire of SUMOylated proteins. In addition, SENP1 regulates the deSUMOylation of the Fragile X Mental Retardation Protein (FMRP) in postsynaptic structures [32]. This is of particular interest in the case of the Fragile X syndrome since the SUMOylation of FMRP is central to the type 1 mGluR-dependent release of its bound mRNAs allowing spine elimination and maturation in the developing brain [9, 33]. In conclusion, the current data provide an additional level of regulation for the SUMOylation process at the mammalian synapse. This is of critical importance since its deregulation may lead to an aberrant composition of SUMOylated proteins at synapses and consequently, participate in the etiology of neurological disorders associated with abnormal type 1 mGluR signaling.

## Materials and methods

### Constructs

GFP-tagged full length WT human SENP1 in pEGFP-C2 is a generous gift from Dr. Wang Min [34]. The construct was entirely sequenced.

### Rat strain

Wistar rats (RRID:RGD_13508588) were from a commercial source (Janvier, St Berthevin, France). Animals were treated in accordance with the European Council Guidelines for the Care and Use of Laboratory animals in our facility. Animals had free access to water and food. Lightning was controlled as a 12-h light and dark cycle and the temperature maintained at 23 ± 1 °C. Protocol to prepare primary neuronal cultures from rat embryos at E18 was approved by the National Animal Care and Ethics Committee (Project reference APAFIS#18647-2019011110552947v3).

### Cell culture

Hippocampal neurons were prepared from E18 pregnant rats as previously described [7, 9, 16, 35]. Briefly, neurons were plated in Neurobasal medium (Invitrogen, France) supplemented with 2% B27 (Invitrogen), 0.5 mM glutamine and penicillin/streptomycin (Ozyme) on 100-mm dishes or 24-mm glass coverslips (VWR) pre-coated with poly-l-Lysine (0.1 mg mL^−1^; Sigma). Neurons (3.10^6^ cells per 100-mm dish or 100,000 cells per coverslip) were then fed once a week with Neurobasal medium supplemented with 2% B27 and penicillin/streptomycin for a maximum of 3 weeks. In all experiments, neurons were preincubated for 10 min in the presence of TTX (0.5 μM) to reduce neuronal activity to basal levels [7, 16, 26, 36] along with the indicated drugs or vehicles.

### Sindbis virus production and neuronal transduction

Attenuated Sindbis viral particles *(SINrep(nsP2S726))* were prepared and used as previously described [37–39]. Briefly, cRNAs were generated from the pSinRep5 plasmid containing the sequence coding for the indicated GFP-SENP1 constructs and from the defective helper (pDH-BB) plasmid using the Mmessage Mmachine SP6 kit (Ambion). cRNAs were electroporated into BHK21 cells. Pseudovirions from the culture medium were harvested 72 h after electroporation and concentrated using ultracentrifugation on SW41Ti. Aliquots of Sindbis particles were stored at -80 °C. Neurons were transduced at 17 to 20 days in vitro (DIV) with a multiplicity of infection (MOI) of 0.05 and returned at 37 °C under 5% CO_2_ for 18 to 24 h until use.

### Immunocytochemistry

Hippocampal neurons (18–21 DIV) were preincubated or not for 10 min in the presence of TTX (0.5 μM) to reduce neuronal activity to basal levels with 0.5 μM JNJ16259685 (Hello Bio) and then treated or not for 30 min with 50 μM DHPG at 37 °C still in the presence of TTX and the indicated inhibitors. Neurons were fixed with methanol for 20 min at − 20 °C and washed three times 5 min in PBS. Cells were then permeabilized for 1 h in PBS containing 0.2% Triton X100, 0.2% of BSA and 5% Horse Serum (HS) at RT. Neurons were stained with a rabbit anti-SENP1 1/200 (Sigma-Aldrich Cat# HPA011765, RRID:AB_1079907), a mouse anti-PSD95 1/500 (Antibodies Incorporated Cat# 75–028, RRID:AB_2292909) and a guinea pig anti-MAP2 1/1000 (Synaptic Systems Cat# 188,004, RRID:AB_2138181) overnight at 4 °C in PBS containing 0.2% Triton X100, 0.2% of BSA. Cells were washed three times in PBS and incubated with the appropriate secondary antibodies conjugated to Alexa488, Alexa594 or Alexa 647 and mounted with Mowiol (Sigma). Confocal images (1024 × 1024 pixels) were acquired with a 63X oil-immersion lens (Numerical Aperture NA 1.4) on a confocal LSM780 microscope (Zeiss, Germany). Z-series of five images of randomly selected dendrites were compressed into two dimensions using the maximum projection algorithm of the Zeiss software (ZEN-ZEISS Efficient Navigation, RRID:SCR_021725). Quantification was performed using the ImageJ software (ImageJ, RRID:SCR_003070) and the extent of synaptic enzymatic colocalisation measured as the Pearson’s correlation coefficient with the JACoP macro.

### Live cell imaging

GFP-SENP1 expressing hippocampal neurons were incubated with the indicated drugs for the time indicated on the figures. A 10-min preincubation at 37 °C was always applied with TTX (0.5 μM) along with the specific inhibitors tested. Pharmacological drugs used in Earle’s buffer (25 mM HEPES-Tris pH 7.4, 140 mM NaCl, 5 mM KCl, 1.8 mM CaCl_2_, 0.8 mM MgCl_2_, 0.9 g L^−1^ glucose) were: TTX (0.5 μM; [7, 16]); JNJ16259685 (0.5 μM); DHPG (50 μM; [7, 16, 40, 41]); Chelerythrine (5 μM; [7, 25]; H89 (1 μM; [26, 42]) and KN93 (1 μM; [27, 43]). Live neurons (18–21 DIV) were kept on a heated stage (set at 37 °C) on a Nikon Ti inverted microscope. GFP fluorescence was excited through a 63X oil-immersion lens (Numerical Aperture, 1.4) using a 488 nm laser light (50 mW, 3%) and time series (0.1 Hz) were collected for a maximum of 70 min as a single image slice using a Perkin Elmer Ultra-View spinning disk microscope. Quantification was performed using the ImageJ software (ImageJ, RRID:SCR_003070).

### Synaptosomal preparation from neuronal cultures

Synaptosomes preparation were adapted from a previously published protocol [7, 15]. Control and stimulated culture of rat cortical neurons at 20 DIV (3 × 60 mm dishes per condition with 6 × 10^5^ neurons/dish) were immediately cooled down on ice after the indicated pharmacological treatments and homogenized in ice-cold sucrose buffer (10 mM Tris–HCl pH 7.4, 0.32 M Sucrose, Mammalian protease inhibitors (Roche) and 20 mM NEM (Sigma-Aldrich) to protect proteins from deSUMOylation). Nuclear proteins were removed from synaptosomal preparation by centrifugation at 1000*g* for 5 min. Post-nuclear fractions were further centrifuged at 15,000*g* for 15 min using a 5424R centrifuge (Eppendorf), to isolate crude synaptosomal fractions. Synaptosomal fractions were lysed directly in 2X Laemli buffer containing 5% *β*-Mercaptoethanol and boiled for 10 min prior to immunoblotting.

### Phospho-PKA and -PKC substrates preparation

Control and stimulated rat cortical neurons at 20 DIV (6 × 10^5^ neurons/60 mm dish) were immediately cooled down on ice after the indicated pharmacological treatments and homogenized in ice-cold sucrose buffer (10 mM Tris–HCl pH 7.4, 0.32 M Sucrose, 1% mammalian protease inhibitors (Roche) and 1% phosphatase inhibitor cocktail (Sigma) to protect proteins from dephosphorylation. Nuclear proteins were removed from cytosolic fraction by centrifugation at 1000*g* for 5 min at 4 °C. Protein concentration in the supernatant was then measured using the Bradford protein assay (Bio-Rad). Samples were then boiled for 10 min in 2X Laemli buffer containing 5% β-Mercaptoethanol.

### Synaptosomal isolation from brain slices

Synaptosomes preparation were adapted from previously published protocols [7, 16, 44]. Three hundred μm-thick P14 rat brain slices were prepared in ice-cold oxygenated (5% CO_2_, 95% O_2_) slicing aCSF solution (125 mM NaCl, 1.25 mM NaH_2_PO_4_, 25 mM Glucose, 1 mM MgCl_2_, 2 mM CaCl_2_, 2.5 mM KCl, 25 mM NaHCO_3_). Free-floating brain slices were first pre-treated with TTX (0.5 μM) in absence or in the presence of JNJ16259685 (0.5 μM) in experimental aCSF solution (119 mM NaCl, 2.5 mM CaCl2, 2.5 mM KCl, 1.25 mM NaH2PO4, 1.3 mM MgSO4, 25 mM NaHCO3, 11 mM Glucose 10 mM HEPES, buffered to pH 7.4) for 10 min at 37 °C and then incubated or not with DHPG (100 µM) for 10 min, still in the presence of TTX and JNJ16259685 as indicated. Brain slices (250 mg) were homogenized in ice-cold sucrose buffer (10 mM Tris pH 7.4, 0.32 M Sucrose, Mammalian protease inhibitors (Roche) and 20 mM NEM (Sigma-Aldrich) to protect proteins from deSUMOylation). Nuclear proteins were removed from the synaptosomal preparations by centrifugation at 1000*g* for 5 min at 4 °C. The supernatant was then loaded on a four-layer Percoll-Sucrose gradient (20%/10%/6%/2% Percoll in sucrose buffer). The gradient was centrifuged at 15,000 rpm for 10 min at 4 °C using a SW41Ti rotor (Beckman). The synaptosomal fraction was recovered at the interface between 10 and 20% and washed in HEPES Buffer (5 mM HEPES pH7.4, 140 mM NaCl, 3 mM KCl, 1.2 mM MgSO_4_, 1.2 mM CaCl_2_, 1 mM NaH_2_PO_4_, 5 mM NaHCO_3_, 10 mM Glucose, mammalian protease inhibitors and 20 mM NEM) and centrifuge at 10,000*g* for 15 min using a JA25.5 rotor (Beckman). The pellet was resuspended in (50 mM Tris–HCl pH 7.5, 150 mM NaCl, 1 mM EDTA, 1% SDS, Mammalian protease inhibitor cocktail 1% and 20 mM NEM) for 1 h at RT. After sonication and centrifugation at 16,000*g* for 15 min using a 5424R centrifuge (Eppendorf), protein concentration in the supernatant was measured using the Bradford protein assay (Bio-Rad).

### Immunoblotting

Protein extracts (25 μg) were resolved by SDS-PAGE, transferred onto nitrocellulose membrane and immunoblotted with the following primary antibodies: Rabbit anti-SENP1 1/250 (Sigma-Aldrich Cat# HPA011765, RRID:AB_1079907); Mouse anti-PSD95 1/10000 (Antibodies Incorporated Cat# 75–028, RRID:AB_2292909); Mouse anti-Homer1 1/1000 (Synaptic Systems Cat# 160 003, RRID:AB_2631222); Mouse anti-Synapsin1a/b 1/1000 (Santa Cruz Biotechnology Cat# sc-376623, RRID:AB_11150313); Mouse anti-SOD2 1/2000 (Santa Cruz Biotechnology Cat# sc-137254, RRID:AB_2191808); Mouse anti-NOPP140 1/700 (Santa Cruz Biotechnology Cat# sc-374033, RRID:AB_10917069); Rabbit anti-Coilin 1/500 (Santa Cruz Biotechnology Cat# sc-32860, RRID:AB_2081431); Rabbit anti-GM130 1/500 (BD Biosciences Cat# 610823, RRID:AB_398142); Rabbit anti-GAPDH 1/10000 (Sigma-Aldrich Cat# G9545, RRID:AB_79620); Rabbit Phospho-(Ser) PKC Substrate 1/1000 (Cell Signaling Technology Cat#2261, RRID:AB_330310); Rabbit monoclonal Phospho-PKA Substrate 1/1000 (Cell Signaling Technology Cat#9624, RRID:AB_331817); Rabbit anti-ERK1/2 1/1000 (Cell Signaling Technology Cat# 4695, RRID:AB_390779); Rabbit anti-phosphoERK1/2 1/2000 (Cell Signaling Technology Cat# 4370, RRID:AB_2315112). Standard loading controls were included using ERK1/2, GAPDH, PSD95 labelling or ponceau staining as indicated.

### Statistical analysis

Statistical analyses were calculated using GraphPad Prism v7.03 (GraphPad Prism, RRID:SCR_002798). Data were expressed as mean ± SEM One-way ANOVA were performed with a Tukey post hoc test for multiple comparison data sets and Ratio paired t-test for the Phospho-ERK experiments. For the immunocytochemistry experiments, Kruskal–Wallis tests were performed with a Dunn’s post hoc test for multiple comparison data sets. All data were tested for normal distribution. *p < 0.05 was considered significant.

### Supplementary Information

Below is the link to the electronic supplementary material.

Supplementary file1 (DOCX 26 KB)

Supplementary file2 (PDF 1276 KB)

Supplementary file3 (AVI 13365 KB)

Supplementary file4 (AVI 10335 KB)

Supplementary file5 (AVI 10372 KB)

Supplementary file6 (AVI 8005 KB)

## Data Availability

Enquiries about data availability should be directed to the authors.
