# Supplementary material for: Bidirectional regulation of synaptic SUMOylation by Group 1 metabotropic glutamate receptors
Source: Cell Mol Life Sci. 2022 Jun 23;79(7):378. doi: 10.1007/s00018-022-04405-z (PMC9226087; doi:10.1007/s00018-022-04405-z)
Supplement: Supplementary file 1 — Supplementary file1 (DOCX 26 KB) [file 18_2022_4405_MOESM1_ESM.docx]

**Supplemental material**

**Bidirectional regulation of synaptic SUMOylation by Group 1 metabotropic glutamate receptors**

### Marie Pronot^1^, Gwénola Poupon^1^, Lara Pizzamiglio^1^, Marta Prieto^1^, Isabel Chato-Astrain, Iliona Lacagne, Lenka Schorova^1^, Alessandra Folci^1^, Frédéric Brau^1^ and Stéphane Martin^2,*^

^1^ Université Côte d’Azur, CNRS, IPMC, France.

^2^ Université Côte d’Azur, INSERM, CNRS, IPMC, France.

*Correspondence to: Stéphane Martin ([martin@ipmc.cnrs.fr](mailto:martin@ipmc.cnrs.fr))

Institut de Pharmacologie Moléculaire et Cellulaire, Centre National de la Recherche Scientifique, UMR7275, Université Côte d’Azur, 660 route des lucioles, 06560 Valbonne, France.

**Supplementary information**

***Supplementary figures***

**Supplementary fig. 1** Control time-lapse recordings on GFP-SENP1-expressing hippocampal neurons.

**a** Representative confocal images of a GFP-SENP1-expressing rat hippocampal secondary dendrite for 70 min at 37°C. Scale bar, 5 µm. **b** Curves represent the mean variation ± SEM of GFP-SENP1 fluorescence in spines (n = 20) and shafts (n = 10) over time. **c** Bar graph shows mean fluorescence intensity ± SEM in spines during the 0-10 min (0.9986 ± 0.001), 10-55 min (0.9991 ± 0.006) and washout (1.004 ± 0.008) incubation periods. Statistics: One-way ANOVA with Tukey post hoc test. P values are indicated on the bars. **Supplementary fig 2** Differential MAPK phosphorylation upon type 1 mGluRs activation.

**a** Representative immunoblotting using anti-phospho- and total ERK antibodies of total proteins extracted from cortical neurons preincubated or not with a mGlu1R antagonist JNJ16259685 (0.5 µM), mGlu5R antagonist MPEP (33 µM) or ERK antagonist PD98059 (1 µM) and treated for 10 min with 100 µM DHPG. ERK total was used as a loading control. **b** Summary histograms show means ± SEM of four independent experiments: [DHPG] (1.690 ± 0.22), [DHPG + JNJ16259685] (1.921 ± 0.31), [DHPG + MPEP] (0.969 ± 0.17), [DHPG + PD98059] (1.380 ± 0.12). Statistics: Ratio paired t-test between treated conditions and the corresponding controls. P values are indicated on the bars. n.s., non-significant. **Supplementary fig. 3** Blockade of mGlu1R activation during type 1 mGluR stimulation leads to a sharp accumulation of SENP1 in a more integrated system.

**a** Step-by-step scheme of synaptosomal isolation. **b** Immunoblots of the different Synaptosomal purification steps with specific markers for the postsynaptic compartments (PSD-95 and Homer1), presynaptic terminals (Synapsin1a/b), the nucleus (Coilin), and the Golgi (GM130). Lanes were labelled as follows: total homogenate (HO), supernatant (S1), pellet 1 (P1), Synaptic Fraction (Syn). Each lane was loaded with 10 µg of proteins. **Supplementary fig. 4** Chelerythrine and H89 prevent the increase in mGluR-induced PKC-dependent and PKA-dependent phosphorylation, respectively.

Cortical neurons (20 DIV) were preincubated for 10 min in TTX in absence (control) or in the presence of Chelerythrin (5µM) or H89 (1 µM) and stimulated in the same medium for 10 min with 50 µM DHPG as described in the Methods section. Cytosolic proteins extracted from the treated neurons were separated by SDS-PAGE and immunoblotted with specific anti-phospho-PKA substrate (**a**) or anti-phospho-PKC substrate (**b**) antibodies. GAPDH is shown as a loading control. **Supplementary fig.5** Kinase inhibition alone or in combination does not modify the subcellular distribution of GFP-SENP1. **a** Curves represent the mean variation ± SEM of GFP-SENP1 fluorescence in spines of GFP-SENP1-expressing rat hippocampal secondary dendrites preincubated in TTX (0.5 µM) for 10 min with either JNJ16259685 (0.5 µM), the PKC antagonist Chelerythrine (5 µM), the CaMKII antagonist KN93 (1 µM) or the PKA inhibitor H89 (1 µM) over time. **b** Curves represent the mean variation ± SEM of GFP-SENP1 fluorescence in spines of GFP-SENP1 expressing hippocampal neurons preincubated in TTX (0.5 µM) and JNJ1659685 for 10 min with either Chelerythrine (5 µM), KN93 (1 µM) or H89 (1 µM) over time. **c** Bar graph shows mean fluorescence intensity ± SEM in spines at the plateau (20-25 min of treatment) in [JNJ16259685] (0.9879 ± 0.009), [Chelerythrine] (0.9954 ± 0.008), [KN93] (1.004 ± 0.007) and [H89] (0.9732 ± 0.034), [JNJ16259685 + Chelerythrine] (1.029 ± 0.020), [JNJ16259685 + KN93] (0.9851 ± 0.013) and [JNJ16259685 + H89] (0.9804 ± 0.021) conditions. Statistics: One-way ANOVA with Tukey post hoc test. P values are indicated on the bars. n.s., non-significant.

**Supplementary video 1** Time-lapse imaging (0.033 Hz; 16 fps) of GFP-SENP1 showing the redistribution of the deSUMOylation enzymes into spines in basal condition and upon mGluRs activation with DHPG.

**Supplementary video 2** Comparative time-lapse imaging (0.033 Hz; 16 fps) of the synaptic GFP-SENP1 fluorescence in basal and JNJ16259685-treated conditions.

**Supplementary video 3** Time-lapse imaging experiments (0.033 Hz; 16 fps) of the synaptic GFP-SENP1 fluorescence variation in basal and [JNJ16259685 + DHPG]-treated conditions.

**Supplementary video 4** Comparative time-lapse imaging (0.033 Hz; 16 fps) of the synaptic accumulation of GFP-SENP1 in [JNJ16259685 + DHPG] ± Chelerythrine, KN93 or H89.
