# Supplementary material for: Bidirectional regulation of synaptic SUMOylation by Group 1 metabotropic glutamate receptors
Source: Cell Mol Life Sci. 2022 Jun 23;79(7):378. doi: 10.1007/s00018-022-04405-z (PMC9226087; doi:10.1007/s00018-022-04405-z)
Supplement: Supplementary file 2 — Supplementary file2 (PDF 1276 KB) [file 18_2022_4405_MOESM2_ESM.pdf]

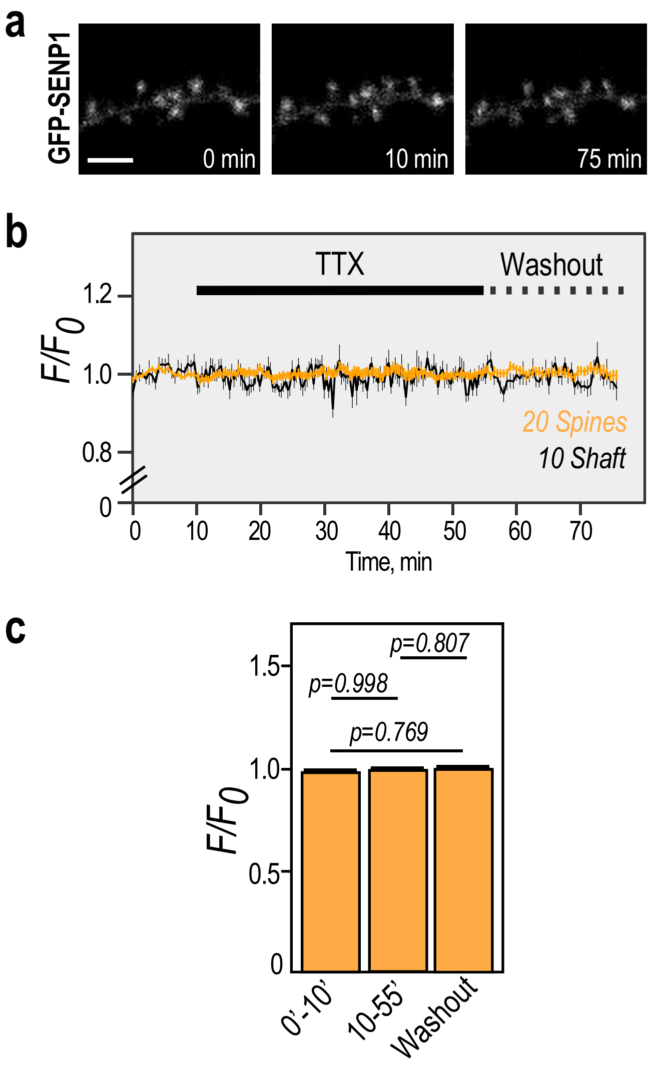

Supplementary Figure 1

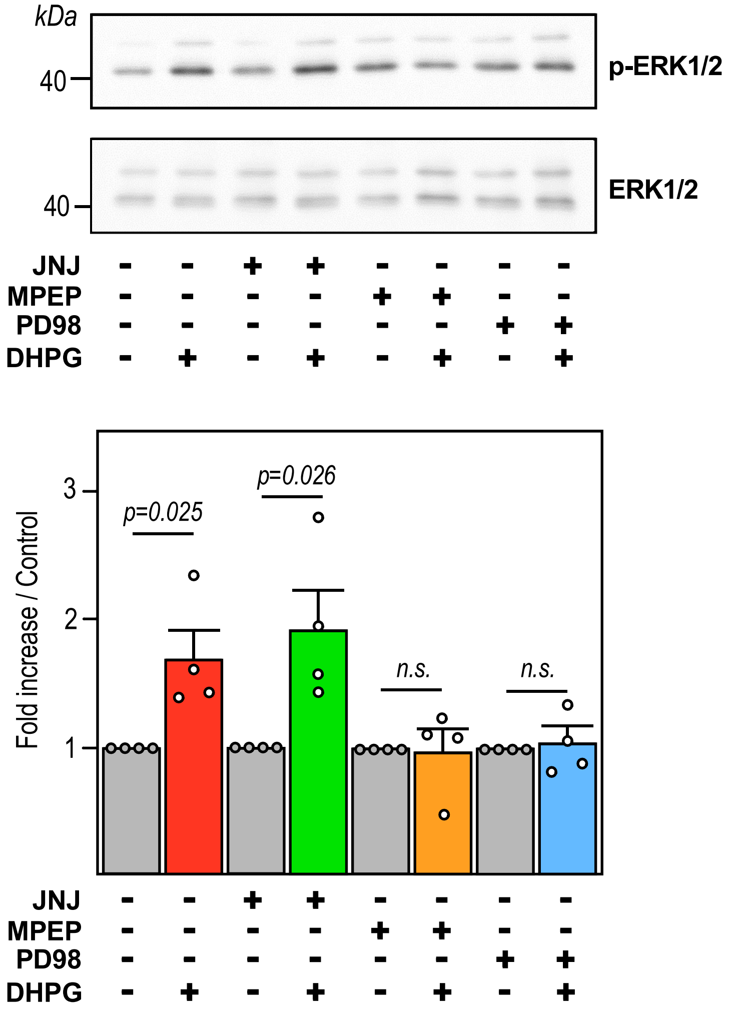

Supplementary figure 2

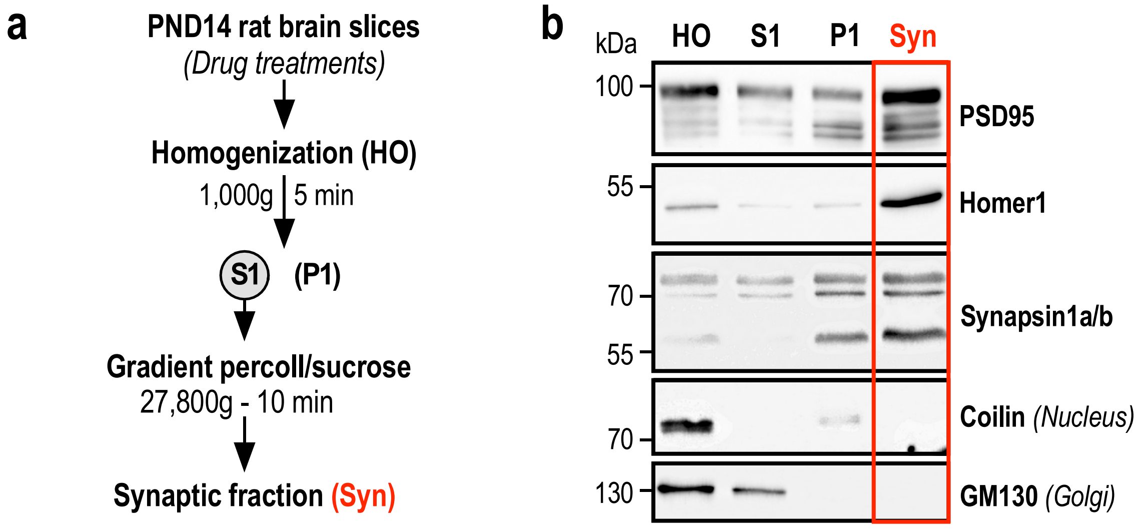

Supplementary figure 3

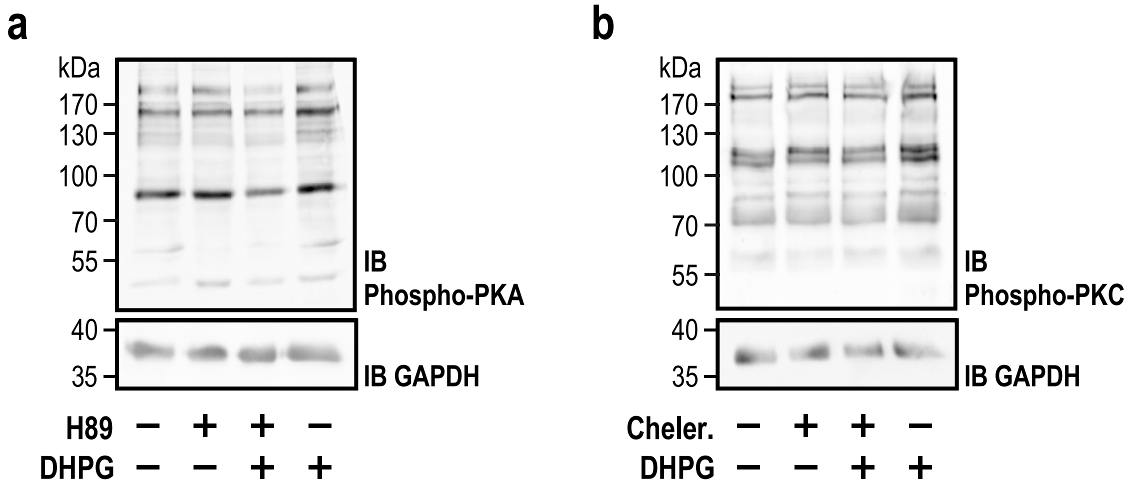

Supplementary Figure 4

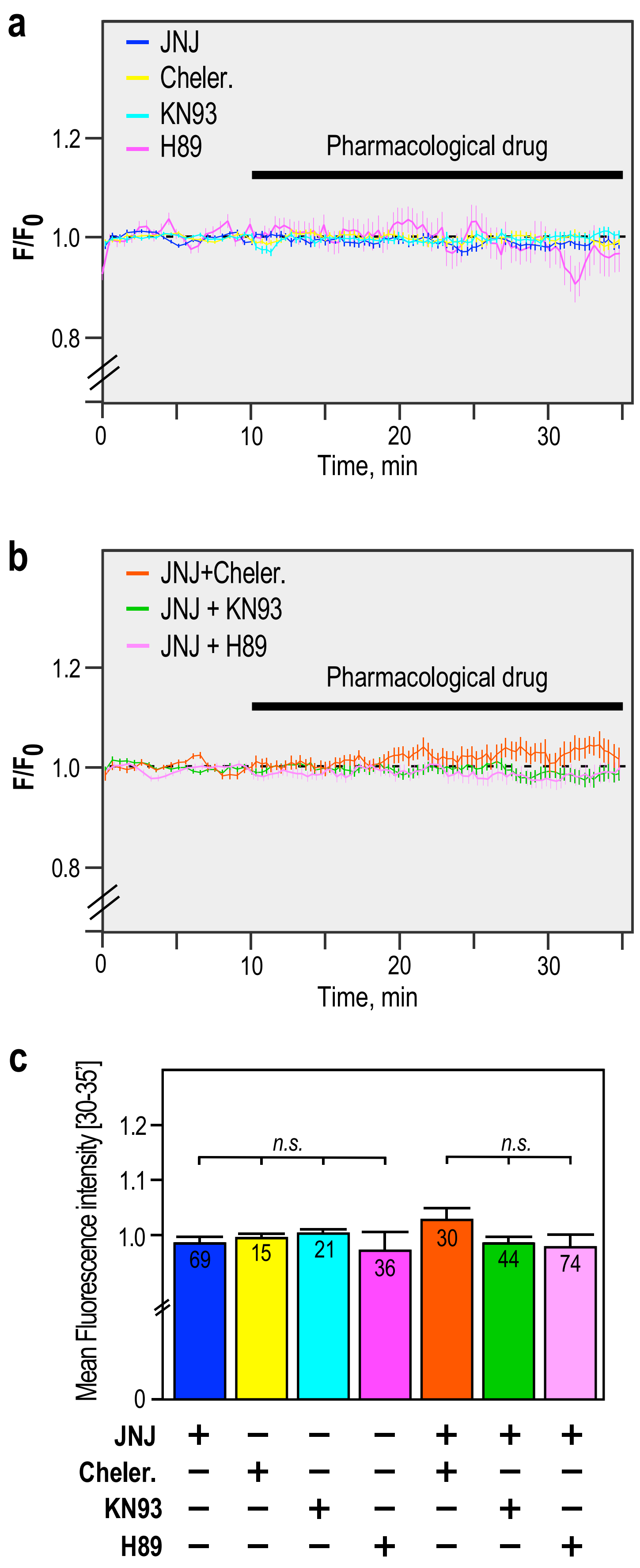

Supplementary Figure 5
